# Nef-Specific CD8+ T Cell Responses Contribute to HIV-1 Immune Control

**DOI:** 10.1371/journal.pone.0073117

**Published:** 2013-09-02

**Authors:** Emily Adland, Jonathan M. Carlson, Paolo Paioni, Henrik Kløverpris, Roger Shapiro, Anthony Ogwu, Lynn Riddell, Graz Luzzi, Fabian Chen, Thambiah Balachandran, David Heckerman, Anette Stryhn, Anne Edwards, Thumbi Ndung’u, Bruce D. Walker, Søren Buus, Philip Goulder, Philippa C. Matthews

**Affiliations:** 1 Department of Paediatrics, Peter Medawar Building for Pathogen Research, University of Oxford, Oxford, United Kingdom; 2 Microsoft Research, eScience Group, Los Angeles, California, United States of America; 3 Department of Immunology and Infectious Diseases, Harvard School of Public Health, Boston, Massachusetts, United States of America; 4 Botswana Harvard AIDS Institute Partnership, Gaborone, Botswana; 5 Department of Genitourinary Medicine, Northamptonshire Healthcare NHS Trust,Northampton General Hospital, Northampton, United Kingdom; 6 Department of Genitourinary Medicine, Wycombe Hospital, High Wycombe, Bucks, United Kingdom; 7 Department of Sexual Health, Royal Berkshire Hospital, Reading, United Kingdom; 8 Department of Genitourinary Medicine, Luton and Dunstable Hospital, Luton, United Kingdom; 9 Laboratory of Experimental Immunology, Faculty of Health Sciences, University of Copenhagen, Copenhagen, Denmark; 10 The Oxford Department of Genitourinary Medicine, the Churchill Hospital, Oxford, United Kingdom; 11 HIV Pathogenesis Programme, the Doris Duke Medical Research Institute, University of KwaZuluNatal, Durban, South Africa; 12 Ragon Institute of Massachusetts General Hospital, Boston, Massachusetts, United States of America; 13 KwaZulu-Natal Research Institute for Tuberculosis & HIV, K-RITH, Nelson R Mandela School of Medicine, University of KwaZuluNatal, Durban, South Africa; 14 Howard Hughes Medical Institute, Chevy Chase, Maryland, United States of America; 15 Nuffield Department of Medicine, Peter Medawar Building for Pathogen Research, University of Oxford, Oxford, United Kingdom; University of Alabama, United States of America

## Abstract

Recent studies in the SIV-macaque model of HIV infection suggest that Nef-specific CD8+ T-cell responses may mediate highly effective immune control of viraemia. In HIV infection Nef recognition dominates in acute infection, but in large cohort studies of chronically infected subjects, breadth of T cell responses to Nef has not been correlated with significant viraemic control. Improved disease outcomes have instead been associated with targeting Gag and, in some cases, Pol. However analyses of the breadth of Nef-specific T cell responses have been confounded by the extreme immunogenicity and multiple epitope overlap within the central regions of Nef, making discrimination of distinct responses impossible via IFN-gamma ELISPOT assays. Thus an alternative approach to assess Nef as an immune target is needed. Here, we show in a cohort of >700 individuals with chronic C-clade infection that >50% of HLA-B-selected polymorphisms within Nef are associated with a predicted fitness cost to the virus, and that HLA-B alleles that successfully drive selection within Nef are those linked with lower viral loads. Furthermore, the specific CD8+ T cell epitopes that are restricted by protective HLA Class I alleles correspond substantially to effective SIV-specific epitopes in Nef. Distinguishing such individual HIV-specific responses within Nef requires specific peptide-MHC I tetramers. Overall, these data suggest that CD8+ T cell targeting of certain specific Nef epitopes contributes to HIV suppression. These data suggest that a re-evaluation of the potential use of Nef in HIV T-cell vaccine candidates would be justified.

## Introduction

The HIV-1 *nef* gene encodes a polymorphic 27kda protein, 200-215 amino acids in length [1,2]. Nef has a complex role in HIV pathogenicity via a number of mechanisms, including down-regulation of host CD4 and MHC cell surface expression, modulation of T cell function, and altering of macrophage signaling [2–4]. Nef is among the most diverse HIV proteins [5]. The greatest sequence variability is focused in the amino- (N-) and carboxy- (C-) terminal regions, while the central portion of the protein is substantially more conserved [6–8]. At least some of this sequence variability may be driven by Class I HLA selection pressure [9,10].

Nef has a high density of overlapping CD8+ T cell epitopes (see Nef epitope map at www.hiv.lanl.gov), and multiple copies of Nef are produced early in the HIV life cycle [11,12]. Nef is the most targeted protein in acute infection [13–15], accounting for 50% to 90% of CD8+ T cell responses in acutely infected subjects [16,17], as well as having the most CD8+ T cell responses and the highest magnitude IFN-gamma responses in chronic infection [7].

Large cross-sectional studies have shown no correlation between viraemic suppression and either the breadth of CD8+ T cell IFN-gamma responses to Nef [18], or the number of HLA-selected mutations in Nef [19]. However the immunogenicity of Nef and consequent density of overlapping epitopes confounds analyses of the distinct CTL responses targeting this protein that have typically depended on ELISpot assays using panels of overlapping peptides 15-18 amino acids in length. Other studies have attempted to address the issue of Nef diversity by using extended panels of overlapping peptides (e.g. 10-mers overlapping by 9 amino acids, with all common variants represented [20]) but this approach is costly and time-consuming.

The HLA Class I alleles that are most strongly associated with long-term suppression of viraemia (e.g. HLA-B*57, -B*58:01 and -B*27 [21–23]) present CD8+ T cell epitopes in Gag and Pol that have been especially well-studied [19,24,25]. The targeting of these highly conserved proteins is thought to underpin successful immunological control [26,27]. However these HLA class I molecules also present epitopes within Nef that may be important in mediating disease control. In a host expressing these protective HLA alleles, the virus adapts by selecting escape mutations in all Gag, Pol and Nef epitopes [28,29], underlining a survival benefit to the virus incurred through evasion of these responses. Indeed, some studies have suggested that viraemic suppression may be linked to specific Nef epitopes, including HLA-B*57/58:01-HW9 [30] and HLA-B*35: 01-VY8 [31].

Several further lines of evidence pointing towards the potential immunological benefit of targeting HIV-Nef have been reported. Many HLA-selected escape mutations in Nef revert to wild-type following transmission to an HLA-mismatched host, suggesting a fitness cost in association with the mutation [9,19]. This hypothesis is supported first by studies showing that Nef polymorphisms are more common among slow progressors than rapid progressors [6], and second by the finding that such sequence changes in elite controllers are associated with a detriment to Nef function [32].

Studies in the SIV model add further weight to the hypothesis that Nef responses can contribute to disease control. In the rhesus macaque, Mamu-B*08, which has a peptide-binding motif so similar to HLA-B*27 that it can bind the same peptides [33], is also associated with elite viraemic control [34–36]. However, unlike HLA-B*27 [25], the immunodominant responses are within accessory/regulatory proteins, and a vaccine induced Mamu-B*08-restricted response to the Nef RL10 epitope (SIV Nef 137–146, RRHRILDIYL) is correlated with viraemic control [34]. This epitope is homologous to the HIV-specific HLA-B*27: 05 RI10 epitope, (HIV Nef 105-114, RRQDILDLWI [37]). Likewise, macaques expressing another favourable allele, Mamu-B*17, also target Nef via two immunodominant epitopes, IW9 (SIV Nef 165-173, IRYPKTFGW) and MW9 (SIV Nef 195-203, MHPAQTSQW) [38,39]. In Mauritian Cynomolgus Macaques, controllers of SIV replication have been shown to target epitopes located near the carboxy terminus of the SIV Nef protein [40]. Recently, a novel protective MHC-I haplotype was described in Burmese macaques, *90-010-Id* (D), which also mediates immune control against SIV through Nef responses [41].

The starting point for these current studies, therefore, was the conflict in evidence regarding the extent to which CD8+ T cell responses to Nef might impact upon HIV-1 disease control. On the one hand, large cross-sectional studies have suggested no benefit in extended breadth of Nef targeting [18,19], whilst on the other, recent data suggest that targeting particular regions of Nef might contribute fundamentally to viraemic control [29–32,34,38,39].

Most previous analyses of Nef diversity and HLA-selection have focused on B-clade infections [12,29,42,43], while studies of C-clade have often been limited by small sample numbers [44,45]. We focused here on an extended cohort of >700 C-clade infected adults to identify relationships between CD8+ T cell responses to Nef and HIV disease control. We hypothesized that certain specific Nef responses may help to underpin viraemic control, strengthening the argument for the inclusion of Nef as an immunogen in CD8+ T cell vaccines.

## Materials and Methods

### Recruitment and characterization of HIV-infected individuals

We studied 739 adults with chronic HIV-1 C-clade infection recruited from three cohorts: Durban, South Africa (n=436), Gaborone, Botswana (n=275), and Zimbabwean subjects recruited via the Thames Valley Cohort in the UK (n=28), as previously described [46,47]. Ethics approval was given by KwaZulu-Natal Review Board and the Massachusetts General Hospital Review Board (Durban cohort); the Office of Human Research Administration, Harvard School of Public Health and the Health Research Development Committee, Botswana Ministry of Health (Gaborone cohort); and the Oxford Research Ethics Committee (Durban, Gaborone and Thames Valley cohorts). All subjects provided written informed consent.

Nef sequences and HLA-types were generated as previously described [19,48]. Viral loads were performed using the Roche Amplicor v.1.5 assay. Viral load measurements were made in chronic disease (therefore reflecting set-point viraemia).

### Identification of Nef sequence polymorphisms associated with Class I HLA genotype

We used computational methods, as previously described [19,49], to identify sites of HLA-selected polymorphism in Nef sequences, correcting for viral lineage and for linkage disequilibrium between HLA Class I alleles. We corrected for multiple comparisons using a q-value (false discovery rate, FDR [50]), and a cut-off value of q<0.05(5% FDR). Reversion was predicted, as previously, by the statistical association between the absence of a particular HLA allele with either (i) an increase in expression of the consensus amino acid, or (ii) a decrease in expression of a variant amino acid [19]. These associations demonstrate that a mutation selected by a particular HLA allele returns to consensus following transmission to an HLA-mismatched host, from which a fitness cost is inferred.

### Data analysis

We computed amino acid variability using Shannon entropy with the on-line tool at Los Álamos HIV sequence database (http://www.hiv.lanl.gov/content/sequence/ENTROPY/entropy_one.html). Mean entropy scores were calculated for each overlapping peptide (OLP) spanning the Nef protein (OLPs 67-93) and ranged from 0.15 in a ‘conserved’ region (OLP-82) to 0.78 in a ‘non-conserved’ region (OLP-68).

Statistical analysis was performed using GraphPad Prism v.5.0a. We focused our attention particularly on the central, most conserved region of Nef (previously defined as residues 81-160 [8]), extended here to correspond to the most conserved overlapping peptides (OLPs) adopted in these and in previously published studies [51], namely OLPs 76-85.

### IFN-gamma ELISpot studies to identify regions of the Nef protein associated with beneficial immune responses

In vitro HIV-specific CD8+ T-cell responses were determined in a cohort of 1010 subtype-C infected individuals using IFN-gamma ELISpot assays using a set of 410 overlapping 18-mer OLPs spanning the whole HIV-1 subtype C proteome (2001 consensus sequence). Overlapping peptides were arranged in a matrix system with 11-12 peptides in each pool. Responses to matrix pools were confirmed by subsequent testing with the individual 18-mer peptides within each pool, and the identity of the individual 18-mers recognized were thus confirmed, as previously described [46].

OLP responses that independently correlate with log_10_ VL were identified using stepwise linear regression (inclusion criterion: p<0.05; exclusion criterion: p>0.05). We included in analysis all OLPs for which responses were observed in ≥3 individuals, and HLA class I alleles expressed in ≥3 individuals. P-values were computed using likelihood ratio tests, and q-values were computed over all features, conditioned on the final model. For these studies, the analyses resulting in q values <0.2 (p<0.024) were defined as significant.

### HLA Class I tetramer staining

Tetramers were generated as previously described [52]. We stained antigen-specific cells using peptide-MHC-I tetramer conjugated to phycoerythrin (PE) or allophycocyanin (APC). Peripheral blood mononuclear cells (PBMCs) were thawed in R10 medium (RPMI medium, 10% fetal calf serum [FCS], 1% penicillin/streptomycin, 1% glutamine); rested for 1 h at 37°C in 5% CO2; stained with HLA class I tetramer for 30 min at room temperature; washed; surface stained with CD8 Pacific Blue (BD Pharmingen), CD3 Pacific Orange (Invitrogen), and a live/dead violet cell stain kit (Invitrogen) for 30 min; washed in phosphate-buffered saline (PBS) and fixed in 2% paraformaldehyde. Data were acquired on an LSR II (BD) flow cytometer within 12 h of staining and analyzed using FlowJo version 8.8.6. The cells were hierarchically gated on singlets, lymphocytes, live cells, and distinct tetramer-specific CD8-positive cells.

## Results

### Over 50% of HLA-selected polymorphisms in Nef are predicted to reduce viral fitness

Sequence analysis identified 44 Nef sequence polymorphisms associated with Class I HLA expression, operating at 34 different amino acid residues (all associations corrected for multiple comparisons, q<0.05 [49]; Table 1). Reversion was predicted following transmission to an HLA-mismatched recipient in 24 of the 44 HLA-associated polymorphisms (54.5%) (Table 1), implying that these polymorphisms would occur at a cost to viral replicative or infective capacity [19,49].

**Table 1:** Amino acid residues in Nef at which amino acid polymorphism is associated with HLA Class I expression.

HLA	HXB2 position																						*P* value	*q* value	Reversion
A*68:01	15	W	S	K	S	S	I	V	G	W	P	**A**	V	R	E	R	I	R	R	T	E	P	5.00E-07	0.00	
A*24:02	135	Q	N	Y	T	P	G	P	G	V	R	**Y**	P	L	T	F	G	W	C	F	K	L	4.40E-05	0.02	R
A*23	143	V	R	Y	P	L	T	F	G	W	C	**F**	K	L	V	P	V	D	P	R	E	V	8.52E-17	0.00	R
A*66:01	156	V	P	V	D	P	R	E	V	E	E	**A**	N	K	G	E	N	N	C	L	L	H	2.87E-05	0.01	R
A*02	176	H	P	M	S	Q	H	G	M	E	D	**E**	D	R	E	V	L	K	W	K	F	D	1.07E-05	0.01	
A*66	184	E	D	E	D	R	E	V	L	K	W	**K**	F	D	S	S	L	A	R	R	H	L	6.70E-05	0.02	R
A*74:01	192	K	W	K	F	D	S	S	L	A	R	**R**	H	L	A	R	E	L	H	P	E	Y	2.89E-06	0.00	R
B*73:01	19	S	I	V	G	W	P	A	V	R	E	**R**	I	R	R	T	E	P	A	A	E	G	1.53E-04	0.05	
B*42	20	I	V	G	W	P	A	V	R	E	R	**I**	R	R	T	E	P	A	A	E	G	V	7.22E-05	0.03	
B*44(03)	64	C	A	W	L	E	A	Q	E	E	E	**E**	V	G	F	P	V	R	P	Q	V	P	2.52E-08	0.00	R
B*4501	64	C	A	W	L	E	A	Q	E	E	E	**E**	V	G	F	P	V	R	P	Q	V	P	3.90E-08	0.00	
B*4501	66	A	W	L	E	A	Q	E	E	E	E	**V**	G	f	P	V	R	P	Q	V	P	L	1.50E-26	0.00	R
B*44(03)	71	E	E	E	E	E	V	G	F	P	V	**R**	P	Q	V	P	L	R	P	M	T	Y	5.23E-06	0.00	R
B*07 [Cw*07:02]	71	E	E	E	E	E	V	G	F	P	V	**R**	P	Q	V	P	L	R	P	M	T	Y	2.16E-13	0.00	R
B*57	76	V	G	F	P	V	R	P	Q	V	P	**L**	R	P	M	T	Y	K	A	A	F	D	5.55E-05	0.02	R
B*81(01)	76	V	G	F	P	V	R	P	Q	V	P	**L**	R	P	M	T	Y	K	A	A	F	D	2.71E-23	0.00	
B*35(01)	81	R	P	Q	V	P	L	R	P	M	T	**Y**	K	A	A	F	D	L	S	F	F	L	8.22E-11	0.00	
B*57	83	Q	V	P	L	R	P	M	T	Y	K	**A**	A	F	D	L	S	F	F	L	K	E	2.41E-07	0.00	
B*5801 [A*03]	83	Q	V	P	L	R	P	M	T	Y	K	**A**	A	F	D	L	S	F	F	L	K	E	1.16E-12	0.00	R
B*44(03)	93	A	A	F	D	L	S	F	F	L	K	**E**	K	G	G	L	E	G	L	I	Y	S	2.48E-15	0.00	R
B*44:03	102	K	E	K	G	G	L	E	G	L	I	**Y**	S	K	K	R	Q	E	I	L	D	L	1.57E-18	0.00	R
B*18(01)	108	E	G	L	I	Y	S	K	K	R	Q	**E**	I	L	D	L	W	V	Y	H	T	Q	1.36E-11	0.00	
B*44:03	108	E	G	L	I	Y	S	K	K	R	Q	**E**	I	L	D	L	W	V	Y	H	T	Q	1.08E-09	0.00	
B*44:03	115	K	R	Q	E	I	L	D	L	W	V	**Y**	H	T	Q	G	F	F	P	D	W	Q	1.25E-04	0.04	R
B*57:03	116	R	Q	E	I	L	D	L	W	V	Y	**H**	T	Q	G	F	F	P	D	W	Q	N	8.93E-06	0.01	
B*35(01)	133	D	W	Q	N	Y	T	P	G	P	G	**V**	R	Y	P	L	T	F	G	W	C	F	3.32E-08	0.00	
B*53(01)	133	D	W	Q	N	Y	T	P	G	P	G	**V**	R	Y	P	L	T	F	G	W	C	F	3.12E-05	0.01	
B*42	133	D	W	Q	N	Y	T	P	G	P	G	**V**	R	Y	P	L	T	F	G	W	C	F	4.75E-05	0.02	R
B*14:01	156	V	P	V	D	P	R	E	V	E	E	**A**	N	K	G	E	N	N	C	L	L	H	5.46E-05	0.02	
B*13	158	V	D	P	R	E	V	E	E	A	N	**K**	G	E	N	N	C	L	L	H	P	M	1.89E-05	0.01	R
B*42	168	K	G	E	N	N	C	L	L	H	P	**M**	S	Q	H	G	M	E	D	E	D	R	1.75E-05	0.01	
B*42	173	C	L	L	H	P	M	S	Q	H	G	**M**	E	D	E	D	R	E	V	L	K	W	4.11E-05	0.02	R
B*15:01	176	H	P	M	S	Q	H	G	M	E	D	**E**	D	R	E	V	L	K	W	K	F	D	1.18E-05	0.01	
B*53(01)	176	H	P	M	S	Q	H	G	M	E	D	**E**	D	R	E	V	L	K	W	K	F	D	2.14E-05	0.01	R
B*08:01	179	S	Q	H	G	M	E	D	E	D	R	**E**	V	L	K	W	K	F	D	S	S	L	1.24E-04	0.04	
B*15:10	191	L	K	W	K	F	D	S	S	L	A	**R**	R	H	L	A	R	E	L	H	P	E	5.99E-06	0.00	R
B*35(01)	194	K	F	D	S	S	L	A	R	R	H	**L**	A	R	E	L	H	P	E	Y	Y	K	1.02E-05	0.01	R
B*42:01	202	R	H	L	A	R	E	L	H	P	E	**Y**	Y	K	D	C							4.76E-05	0.02	
Cw*02:10	48	K	H	G	A	L	T	S	S	N	T	**A**	H	N	N	A	D	C	A	W	L	E	1.51E-04	0.05	
Cw*08	85	P	L	R	P	M	T	Y	K	A	A	**F**	D	L	S	F	F	L	K	E	K	G	3.19E-05	0.01	R
Cw*07:01 [B*080	105	G	G	L	E	G	L	I	Y	S	K	**K**	R	Q	E	I	L	D	L	W	V	Y	2.93E-14	0.00	R
Cw*16:01	174	L	L	H	P	M	S	Q	H	G	M	**E**	D	E	D	R	E	V	L	K	W	K	1.13E-04	0.04	
Cw*16	198	S	L	A	R	R	H	L	A	R	E	**L**	H	P	E	Y	Y	K	D	C			1.26E-05	0.01	R
Cw*16	199	L	A	R	R	H	L	A	R	E	L	**H**	P	E	Y	Y	K	D	C				1.44E-05	0.01	R

Bold interface indicates site of polymorphism

Lineage-corrected analysis of sequences from 739 C-clade infected Southern African subjects. Amino acid residue at which polymorphism is identified is shown in bold, with ten amino acids flanking on either side (consensus sequence for this cohort). Known HLA-class I epitopes corresponding to the allele in question are underlined. Sites at which reversion is predicted are marked ‘R’ [19]. Round brackets indicate likely high resolution HLA type based on high population prevalence of this allele. Square brackets indicate alleles in linkage disequilibrium with selecting allele, with association also detected at this site.

Of the 44 HLA-associated polymorphisms identified, 25 (57%) were within or in the flanking regions (within 5 amino acids up- or down-stream) of known epitopes restricted by the HLA allele in question (Table 1). The majority of polymorphisms (n=31, 70%) were associated with HLA-B alleles, while 7 (16%) were associated with HLA-A and 6 (14%) with HLA-C (Table 1).

### HLA-mediated selection pressure contributes to sequence diversity in conserved central Nef

**Figure 1:**
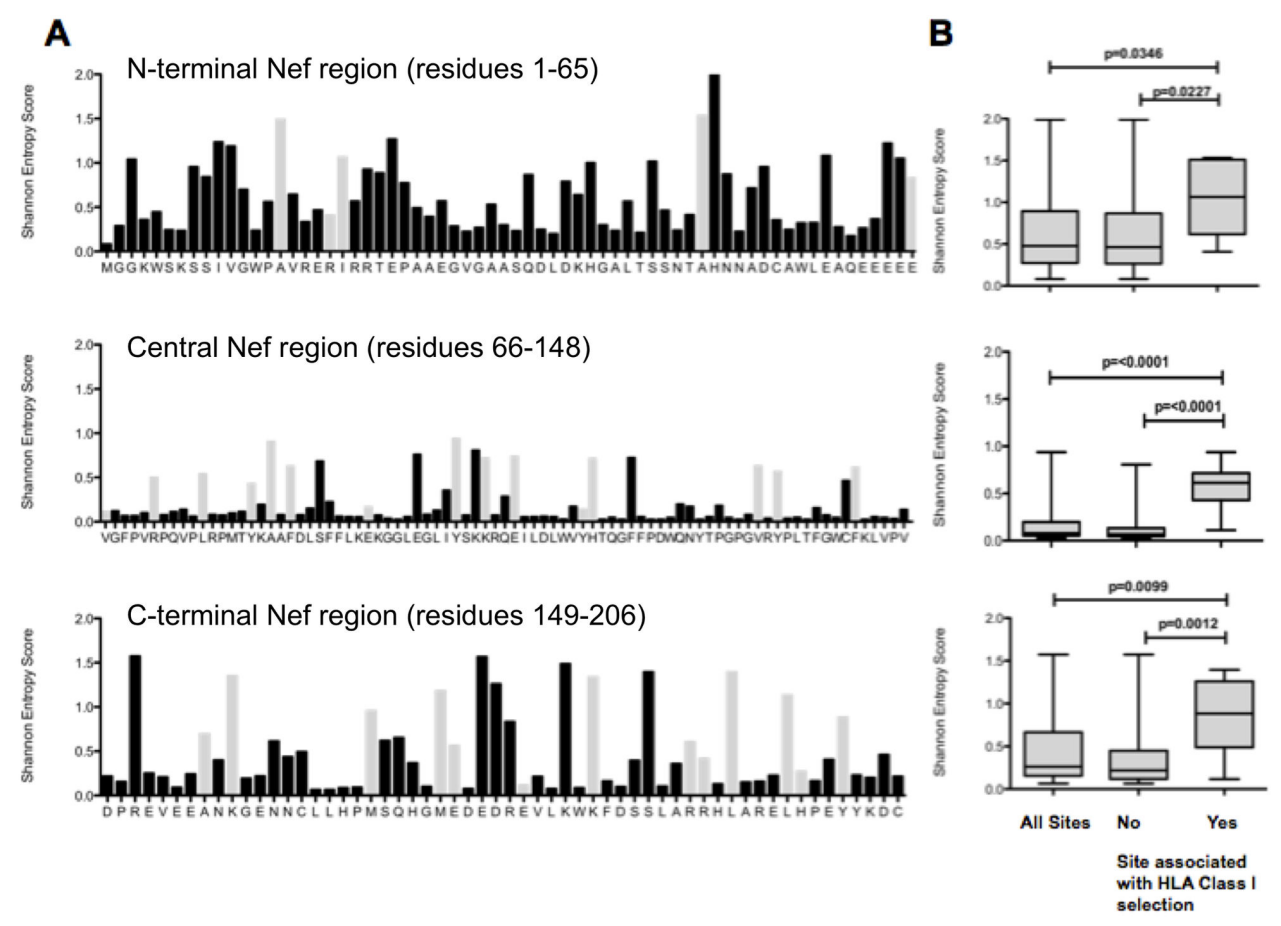
Variation in C-clade Nef sequences, and relationship between amino acid variability and presence of HLA-class I selection pressure. [A] Nef consensus sequence (derived from 739 C-clade sequences from Southern African patients) plotted against Shannon entropy score. Residues at which there is an association with HLA-Class I expression are shown in grey. As previously observed, the sequence is most highly conserved in the central portion of the protein (residues 66-148) [8].. [B] Entropy scores of residues associated with HLA-class I expression vs. those with no HLA association, showing significantly higher variability at sites at which HLA selection operates, particularly in the central conserved region. P-value by Mann Whitney U test.

Nef can be divided into three regions of differing sequence variability (Figure 1). The central region (residues 66-148, median residue entropy 0.135) is the most conserved, significantly more so than both the C-terminal region (residues 149-206, median residue entropy 0.491, p < 0.0001, Mann–Whitney U test), and the N-terminal region (residues 1-65, median residue entropy 0.829, p < 0.0001, Mann–Whitney U test). The C-terminal region is significantly more conserved than the N-terminal region (p=0.0056, Mann–Whitney U test).

There is a strong relationship between the presence of an HLA-selected polymorphism and amino acid variability, particularly in the central conserved region of Nef (p=0.0001, Mann–Whitney U test; Figure 1B). Although this type of analysis cannot determine the causation of amino acid diversity, this association may indicate that HLA is a significant factor in driving polymorphisms, as previously suggested with Gag sequences [10] and supported in studies of longitudinally sampled Nef sequences [53]. This illustrates the potential evolutionary influence of HLA-selection in Nef [29,54], 47], as a factor contributing to sequence diversity, together with other factors such as neutral ‘toggling’ between amino acids, founder effect and genetic drift [55].

### HLA-B selected mutations in Nef are associated with reduction in viral set-point

**Figure 2:**
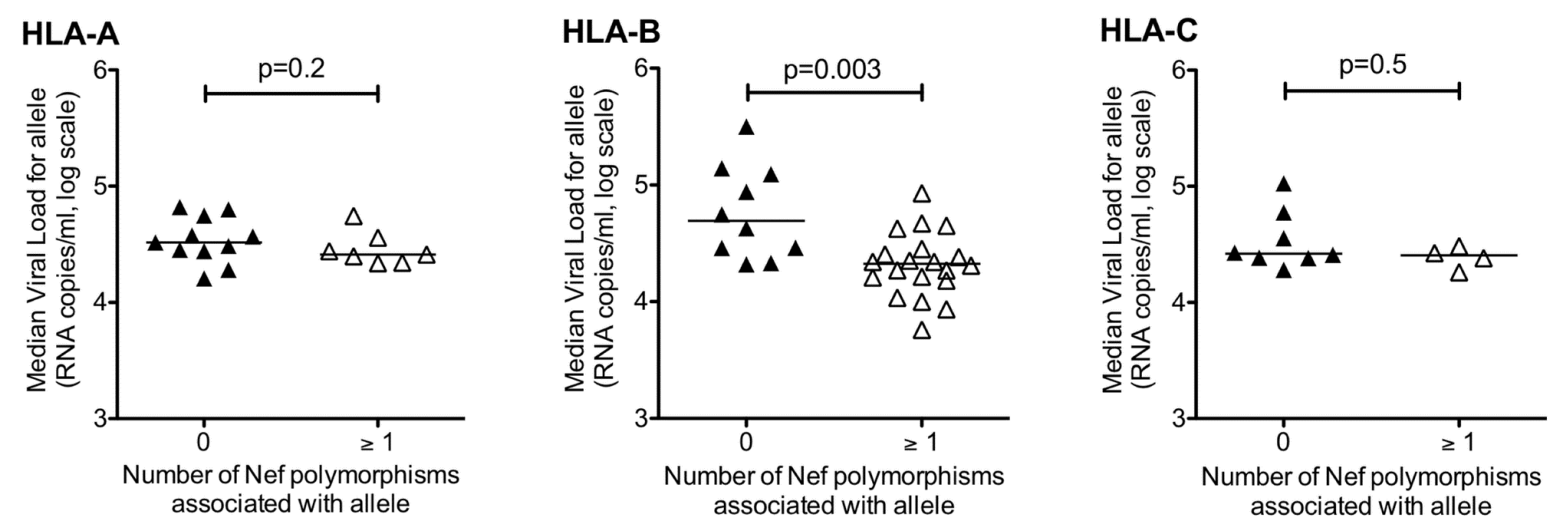
Relationship between number of HLA associations with Nef sequence polymorphisms and median viral load for subjects expressing that allele, for HLA-A, -B and -C alleles. Data from lineage-corrected analysis of 739 C-clade Nef sequences. P-values by Mann–Whitney U test.

We next investigated the relationship between the presence of polymorphisms selected by HLA-A, HLA-B and HLA-C alleles and viral load (VL) in chronic infection. HLA-B alleles driving the selection of Nef mutants are linked with lower VL than those that do not select Nef polymorphisms (median VL 21,175 RNA copies/ml versus 49,350 for HLA-B alleles not associated with any Nef selection; p=0.003, Mann Whitney U test; Figure 2). However, increasing the number of Nef mutations beyond 1 was not statistically associated with HLA-B alleles predictive of lower VL. There was no significant relationship between the selection of mutations and median VL for HLA-A and HLA-C alleles (p=0.2 and p=0.5, respectively, Figure 2).

Given the relationship between HLA-B selection in Nef and reduced VL, we further scrutinized the pattern of HLA-B-associated polymorphisms. The commonest HLA-B alleles driving selection were HLA-B*44 (n=6; 14% of all associations), HLA-B*42 (n=5; 11%), HLA-B*57 (n=3; 7%) and HLA-B*35 (n=3; 7%) (Table 1). All of these alleles have been associated with favourable control of viraemia in previous studies of C-clade infected cohorts [31,56–58]. This includes HLA-B*35: 01, which is associated with rapid progression in B-clade infection but not in C-clade infection [31]. The presence of a strong CD8+ T cell response directed at Nef by all of these alleles is again consistent with the hypothesis that specific Nef responses may be a component of successful disease control.

Definition of novel HLA-B*44:03 restricted Nef epitopes

**Figure 3:**
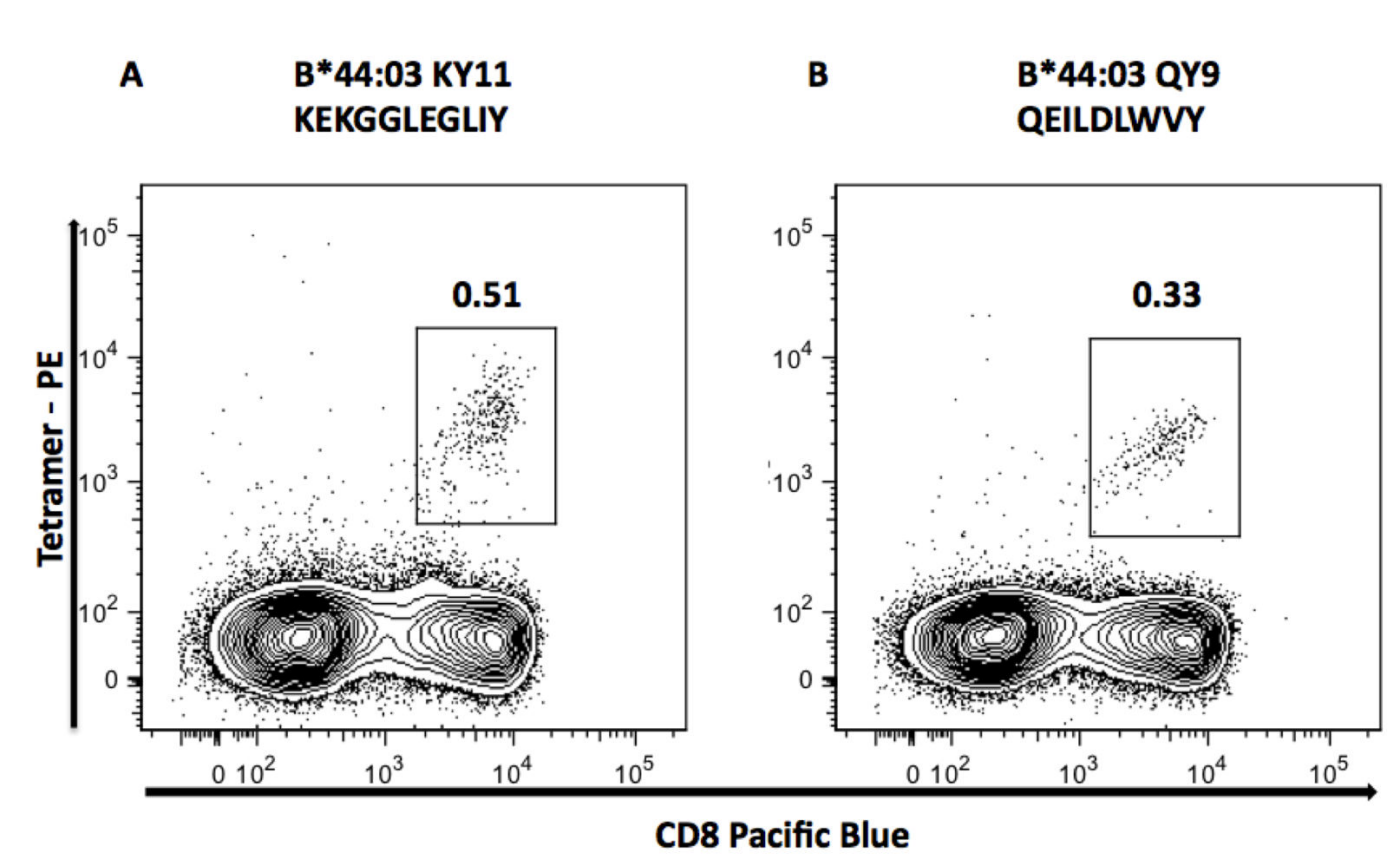
Confirmation of Nef-KY11 (KEKGGLEGLIY) and Nef-QY9 (QEILDLWVY) as novel HLA-B*44: 03 restricted CD8 T cell **epitopes using** HLA-Class I tetramers. HLA-B*44: 03 tetramers were loaded with [A] Nef-KY11 and [B] Nef-QY9 optimal peptide and stained against donor PBMC’s from a C-clade infected B*44: 03 positive individual, subject R129 HLA-A*23: 01/30:04, B*08: 01/44:03, C*03:04/04:01. The number shown above each gate is the percentage of total lymphocytes that are tetramer-specific (tetramer positive cells expressed as a percentage of CD8+ cells were 1.48% for KY11 and 0.65% for QY9).

**Figure 4:**
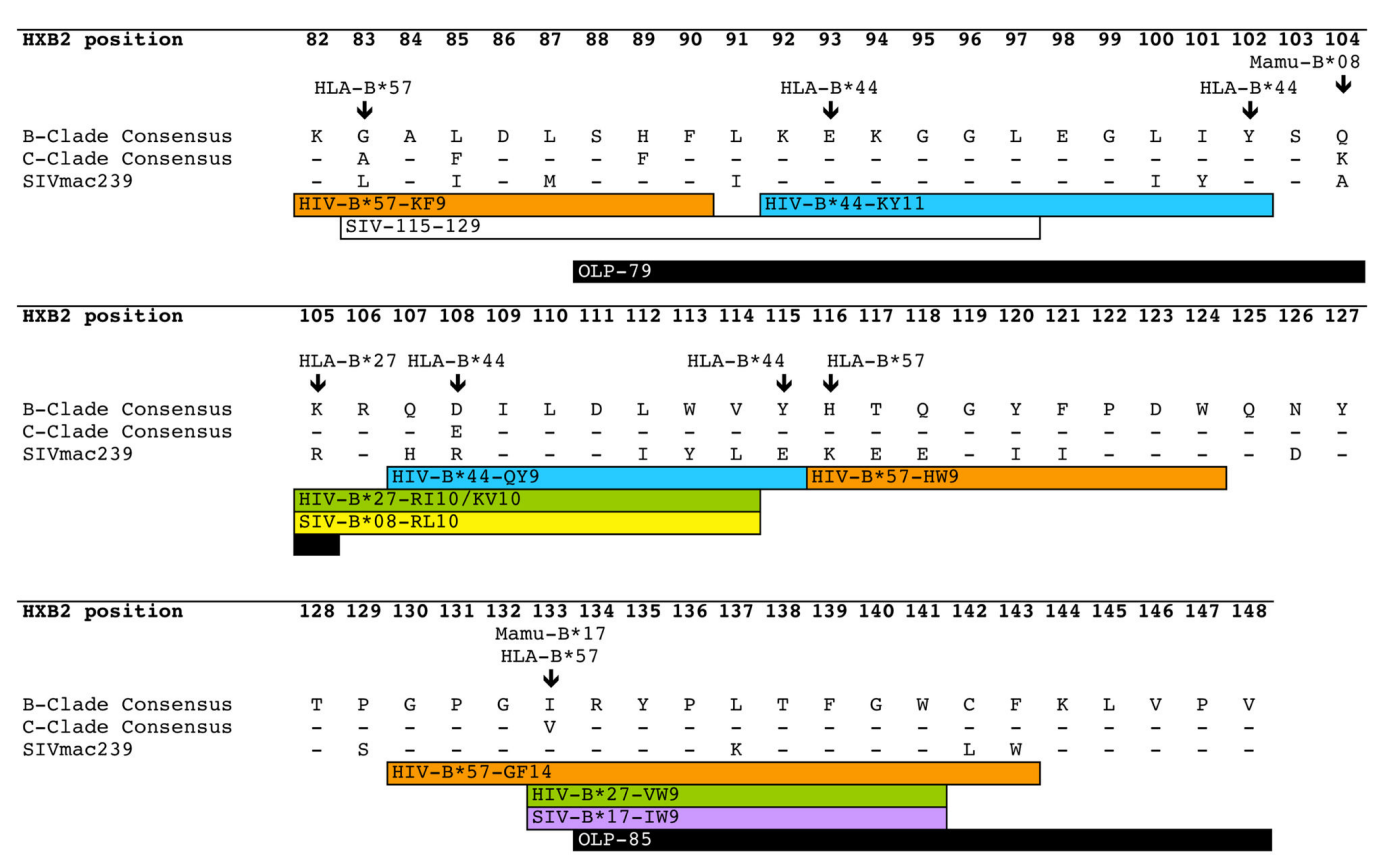
Map of central region of Nef showing sites of key epitopes and residues at which CD8+ selection pressure operates. Central conserved region of HIV-1 Nef is shown previously defined as HXB2 residues 81-160 [8]. Corresponding B-clade and C-clade consensus sequences are shown along with SIVmac239 consensus. Positions of epitopes restricted by alleles HLA-B*27 [37], HLA-B*57 [28,64,65] and HLA-B*44 are highlighted in green, orange and blue respectively. Regions homologous to Mamu-B*08 [36] and Mamu-B*17 epitopes [66] are also marked (yellow and purple respectively). SIV 115-129 also highlighted as a region recently associated with SIV control in macaques [41]. Responses to overlapping peptides 79 and 85 are associated with viraemic control, q<0.2 (black boxes). Sites of mutations selected by HLA-B*57, HLA-B*27, HLA-B*44, Mamu-B*08 and Mamu-B*17 are marked with arrows [29,36,67]. This highlights the substantial overlap between HIV and SIV epitopes restricted by alleles that are associated with favourable immune control.

HLA-B*44 is responsible for the greatest number of HLA-selected polymorphisms in Nef (Table 1), but there are no epitopes restricted by HLA-B*44 that have been sufficiently well defined to appear in the Los Álamos epitope ‘A-list’ (http://www.hiv.lanl.gov/content/immunology/tables/optimal_ctl_summary.html). In Southern Africa, the predominant HLA-B*44 subtype is HLA-B*44: 03. For these reasons, we focused on further identification of HLA-B*44: 03 epitopes. The approach we used for guidance was, first, to identify the Nef residues at which HLA-B*44: 03 drives escape, then to predict the optimal epitope based on the peptide-binding motif for HLA-B*44: 03, which features glutamate (E) at position 2, and phenylalanine (F) or tyrosine (Y) at the C-terminus [59,60]. We thus identified Nef 93-102 (KEKGGLEGLIY; locations of HLA-escape associations underlined) and Nef 107-115 (QEILDLWVY) as putative HLA-B*44: 03-restricted epitopes. We subsequently confirmed these epitopes using HLA-B*44: 03 tetramers bound to KY11 or QY9 (Figure 3). In both epitopes, the putative escape mutations are located exclusively at the anchor residues, consistent with recent studies indicating a tendency for selection of escape at anchor residues [61], especially among protective alleles. The location of these epitopes, which overlap with others presented by highly protected MHC-I alleles (Figure 4), as well as with many others within the central highly immunogenic region of Nef (see epitope map at http://www.hiv.lanl.gov/content/immunology/maps/ctl/Nef.html) would make precise identification, quantification and analysis of such responses highly problematic without access to such tetrameric reagents.

### CD8+ T cell responses to certain Nef regions associated with lower viral load set-point

Our analyses of this extended dataset confirmed previous findings [51] of a statistically significant relationship between reduced VL and IFN-gamma ELISpot T cell responses to two OLPs in Nef, with q<0.2: OLP-79 SFFLKEKGGLEGLIYSKK (p=0.01, q=0.13), and OLP-85, RYPLTFGWCFKLVPV (p=0.002, q=0.04).

Examining these regions in more detail, the Nef regions spanned by OLP-79 and OLP-85 contain epitopes restricted by HLA-B*27, HLA-B*44 and HLA-B*57 [28] (http://www.hiv.lanl.gov/content/immunology/ctl_search and [14]) as well as by the protective macaque MHC-I alleles Mamu-B*08 and Mamu-B*17 and the protective Burmese rhesus monkey haplotype *90-010-Id* (D) (Figure 4). Together, these data suggest that responses to these regions of Nef may be especially beneficial and contribute to MHC-I-associated protection against HIV/SIV disease.

## Discussion

We have here shown, using several approaches, that CD8+ T cell responses to HIV Nef can be linked with improved viraemic suppression. First, HLA-selected escape mutations in Nef have a high rate of reversion to wild-type following transmission, suggesting a fitness cost imposed by the mutation in the presence of the selecting allele. Second, targeting of two specific OLPs in Nef is associated with reduction in viraemia in chronic infection, pointing to successful T cell responses in these regions. Third, there is substantial overlap between Nef regions targeted by beneficial HLA Class I epitopes and by simian Mamu-restricted responses that are associated with disease control.

The finding that HLA alleles predictive of lower viraemia are statistically associated with selection of one or more HLA-selected polymorphisms in Nef points to a contribution of CD8+ T cell responses to Nef in mediating disease control. It is problematic however to infer the impact of individual mutations by comparison of viral loads in subjects with and without those mutants. If the mutation is associated with a significantly increased viral load this may suggest that the CTL response contributes to immune control and if there is no significant increase that it does not. However, a lack of significant increase in viral load associated with selection of the escape mutant may reflect reduced replicative capacity resulting from that mutation and a fine balance between selection pressure driven by the CTL response and opposing purifying selection pressure exerted by the virus. The fact that 5 out of the 6 B*44: 03-associated Nef mutations were predicted to be revertants, that is, induced a cost to viral replicative capacity, underlines the intractability of such analyses. For these reasons, we cannot draw specific conclusions about the effects of selection of one or more Nef polymorphisms; however, these results do suggest that the ability to target Nef epitopes with sufficient strength that escape associations can be detected population-wide is a correlate of protection.

HLA-B*44 is most frequently associated with sequence polymorphism within Nef of any HLA allele and is also consistently associated with some tendency to protection against disease progression or control of viraemia, amounting to approximately a 0.25 log_10_ reduction in viral set point on average, compared to a ~0.75 log_10_ reduction for HLA-B*57: 03, the most protective HLA allele prevalent in sub-Saharan African populations [31,46,56]. We have here defined two novel HLA-B*44: 03 restricted epitopes within Nef, KY11 (KEKGGLEGLIY) and QY9 (QEILDLWVY) (Figure 3). KY11 is entirely contained within OLP-79 which has been associated with improved disease control overall. Due to the small number of HLA-B*44: 03 responders to this OLP in our cohort (2 responders of a total 91 subjects with HLA-B*44: 03), we have been unable to determine whether recognition of the KY11 epitope itself contributes to control of viraemia. These tetramer-based confirmations of the optimal epitopes in this central region of Nef highlight the necessity to use these reagents in order to characterize CTL responses that are potentially critically important to immune control.

The overall degree of sequence diversity in Nef might suggest that amino acid substitutions would be well tolerated without a significant detriment to viral infectivity or replicative capacity. However, a reversion rate of >50% (Table 1) suggests that, even in variable regions of the protein, a fitness cost to the virus is commonly imposed by the selection of HLA escape mutations. The polymorphisms we identified do not only occur in the epitope-rich central portion of Nef, but also in the terminal regions of the protein, particularly at the C-terminal region (Figure 1). This finding suggests that the C-terminal region of Nef as well as the central, highly conserved region, also contains CD8+ T cell epitopes. Potentially these may also be important, but the more variable regions of the viral proteome present a considerable challenge to epitope definition and characterization.

It is striking that SIV epitopes in Nef that are associated with immune control correspond so specifically to sites of confirmed or putative HIV HLA-B*57 and B*27 epitopes, and with HIV-OLPs that are associated with disease control (Figure 4). The substantial overlap between favourable responses in SIV and HIV highlights the utility of SIV studies in detecting regions of the HIV proteome that warrant further attention. However it is also notable that sequence conservation is not the only factor contributing to the potential efficacy of Nef-specific responses: disease protection was only associated with responses to OLPs-79 and -85, while responses to OLPs 80-84 which span this region - and are equally conserved and just as immunogenic as OLP-79 and OLP-85 - are not associated with lowered VL (Figure 4). This observation is also highlighted by other recent studies that report substantial variation in fitness costs associated with HLA-selected mutations irrespective of the degree of sequence conservation [62,63].

Overall, these results confirm the importance of Nef as a CD8+ T cell target with a potentially significant impact on HIV viraemic control, highlight the potential for HLA selection to drive evolutionary change, and corroborate the predominance of the HLA-B locus as a selection force. The high rate of reversion suggests that over half of the HLA selected polymorphisms are associated with a significant cost to viral fitness, underlining the usefulness of this protein as a vaccine target. Further studies to characterize the functional role of these regions of Nef and to define more CD8+ T cell epitopes will further inform the use of Nef as a vaccine immunogen.
